# An analysis of child deaths by suicide in Queensland Australia, 2004-2012. What are we missing from a preventative health services perspective?

**DOI:** 10.5249/jivr.v9i2.837

**Published:** 2017-07

**Authors:** Florin Oprescu, Bridie Scott-Parker, Jeanne Dayton

**Affiliations:** ^*a*^School of Health and Sport Sciences, Faculty of Sciences, Health, Education, and Engineering, University of the Sunshine Coast, Queensland, Australia.; ^*b*^Adolescent Risk Research Unit (ARRU), School of Social Sciences, Faculty of Arts and Business, University of the Sunshine Coast, Queensland, Australia.; ^*c*^Sustainability Research Centre (SRC), Faculty of Arts and Business, University of the Sunshine Coast, Queensland, Australia.; ^*d*^Sunshine Coast Mind and Neuroscience – Thompson Institute, University of the Sunshine Coast, Queensland, Australia.

**Keywords:** Suicide, Children, Adolescents, Risk factors, Prevention

## Abstract

**Background:**

This article analyzes case descriptions of child suicides from 2004 to 2012 to inform future policy and practice.

**Methods:**

Quantitative data and case descriptions for 159 child suicides (less than 18 years) in Queensland, Australia, were analyzed quantitatively using SPSS and qualitatively using automated content analysis (Leximancer).

**Results:**

More than three quarters of child suicides involved hanging and 81% of suicides occurred in the family home. Less than 20% of the deceased left a note, however there was evidence of planning in 54% of cases. Most common triggering events were family conflicts.

**Conclusions:**

Effective suicide prevention interventions require a comprehensive understanding of risk factors. Quality of case descriptions varied widely, which can hamper injury prevention efforts through an incomplete understanding of characteristics of and important factors in child suicide. Additional attention and resources dedicated to this public health issue could enhance the development and implementation of effective intervention strategies targeting child and adolescent suicide.

## Introduction

Youth and child suicide prevention remains a priority across the globe and in Australia,^1^ with suicide representing 26% of deaths in Australia and being the most common cause of death in the 15-24 years population. ^2^ The National Youth Suicide Prevention Strategy imple-mented in 1999 has been instrumental in reducing ado-lescent suicide. ^1,3^ However, the Queensland rate, 2.34 per 100,000 5-14 year olds over four years (2008-2012), remains high compared to the Australian average of 2.03 per 100,000. ^4,5^ Thus an analysis of this data could provide additional insights for prevention. 

Suicide risk factors should be considered in the context of a broader framework of environmental risk and protective elements, taking into account social, economic and cultural stressors.^6-9^ McNamara ^8^ in particular provides an extensive list of risk factors, separating individual, family and community level factors, emphasizing the inherent interactions between each level and the significance of family problems in particular. Research associates problems with school, romantic-partners, friends, family, and impending or recently past crises with child suicide. ^10^ There appears to be similarities in mental health difficulties amongst youth both with suicidal ideation and with previous suicide attempts, ^10,11^ with 25% of 16-24 year olds reporting a mental health disorder in the past 12 months and 40% at any time prior to this period. ^12^

Theories of suicidal behavior incorporate some of the aforementioned elements and provide directions for research and prevention. Examples include the interpersonal-psychological theory of suicidal behavior,^13-15^ which identifies desire and capability as predictors for suicide, with desire being influenced by beliefs of being a burden and social isolation. ^16^ Klonsky and May identified impulsivity as a critical factor for suicide attempts, ^17^ and proposed the three-step theory of suicide which considers psychological pain, isolation and capability as predictors of suicide attempts and areas for intervention. ^18^ O’Connor proposed a three phase model of suicidal behavior ^19^ and identified pre-existing vulnerabilities such as hopelessness, impulsivity, perfectionism and neuroticism as critical factors for suicide attempts. ^20^

In relationship vulnerability, in addition to males being at higher risk, within the Australian context the risk of suicide is also increased for persons who are Indigenous, live in rural areas, are refugees or recently immigrated, same-sex attracted, transgender, have a lower socio-economic status and those who are involved in the welfare system or in residential care.^7,8,21-23^ There are documented links between social support and mental health ^24^ that may increase the risk in these populations. For example, diminished social capital ^25^ coupled with poor mental health literacy and a reluctance to seek help, which are prevalent in young populations, can considerably increase the suicide risk. ^8^ In addition, sexual abuse and substance abuse have been found to increase risk of suicide attempts. ^26^ Furthermore, children under 15 years are considered to lack the capacity to understand the irreversibility of death. ^8^

**Study aims**

Both quantitative and qualitative research into child suicide could be used to inform future research, policy and practice,^21,27^ therefore this article aims to analyze the available case descriptions of Queensland child suicides from 2004 to 2012, thus identifying contributors to and risk factors for child suicide. The study methodology was approved by the University Ethics Committee.

## Methods

De-identified data pertaining to Queensland child fatalities (<18 years) resulting from suicide during the period 1 July 2004 to 30 June 2012 was obtained from the Commission for Children and Young People and the Child Guardian (CCYPCG) in Queensland in the form of demographic variables and case descriptions. To be classified as suicide, either the child’s intent was clearly established or a probable intention to die was established from coronial, police, health, and/or education records. 

Quantitative case data available was analyzed using the Statistical Package for Social Sciences (SPSS) Version 22 for all cases. Descriptive statistics of frequency of occurrence of each factor available for analysis was reported alongside inferential analysis between categorical variables (i.e., age category, gender, Aboriginal and Torres Strait Islander status, and method of suicide, against other variables).

An exploratory content analysis using Leximancer Version 4 coding of a complete data set (n=159). Default text processing and concept seed settings were used, as they were suitable to the data. A review of concepts generated led to the removal of redundant concepts such as time, down, and approximately that contained minimal specificity. Similar concepts such as house, home, and residence; deceased and deceased’s; young and person, were combined. Case data (n=159) in the form of narrative summaries of the circumstances surrounding the death supplied to the researchers varied widely in terms of the detail provided, from single sentence to 400 word descriptions.

## Results

**Quantitative analysis**

Quantitative analysis (n=159) in Table 1 included an analysis of frequencies of demographic, method of death, and potential precipitative factors. Subsequently, associations between each variable were explored, with significant and non-significant trends reported next. There was a non-significant trend (p=0.066) towards a higher percentage of people who identified as Aboriginal in the younger age group of 9-14 year olds. There was also a non-significant trend (p=0.057) towards alcohol, drug or substance use as a precipitating factor in the older age category of 15-17 year olds. The older group were similarly more likely to have stated or implied their intent to suicide (p=0.089) and left a suicide note (p=0.096). 

**Table 1:** Descriptive statistics of child suicides in Queensland, Australia 2004-2012 (n=159).

Demographic factors		
		Percent
Age	15-17 years	76.1
	9-14 years	23.9
Gender	Female	36.5
	Male	63.5
ARIA Of Usual Residence	Major Cities	40.3
	Inner Regional	24.5
	Outer Regional	20.8
	Remote	2.5
	Very Remote	10.7
	Outside of Queensland	1.3
SEIFA of usual Residence	Very high	7.5
	High	15.7
	Moderate	29.6
	Low	25.8
	Very low	20.1
	Outside of Queensland	1.3
Aboriginal and Torres Strait Islander	Aboriginal	23.9
	Torres Strait Islander	1.9
	Neither	73.6
	Unknown	.6
Known to child safety system with-in 3 years of death	No	63.5
	Yes	36.5
		
	Method Of Death	
Method Of Death	Asphyxiation/inhalation of gases	.6
	Carbon monoxide poisoning	1.9
	Drug overdose	1.9
	Fall from height	.6
	Gunshot wound	4.4
	Hanging/strangulation	81.1
	Jumped from a height	1.3
	Jumping in front of moving object	5.0
	Lying in front of a moving object	1.3
	Motor vehicle collision	1.3
	Self-immolation	.6
		
	Mental Health And Behavioral Issues	
Mental Health And Behavioral Issues	No	41.5
	Unknown	5.0
	Yes	53.5
More Than One Mental Health Or Behavioral Issue Identified	No	76.7
	Unknown	6.3
	Yes	17.0
Family History Of Mental Illness	No	58.5
	Unknown	25.2
	Yes	16.4
		
	Previous Suicidal Thoughts And/Or Behavior	
Previous Suicidal Thoughts And/Or Behavior	No	39.0
	Unknown	2.5
	Yes	58.5
Attempted Suicide	No	68.6
	Unknown	5.0
	Yes	26.4
Self Harm	No	62.9
	Unknown	9.4
	Yes	27.7
Suicidal Ideation	No	54.1
	Unknown	5.7
	Yes	40.3
		
	History Of Childhood Abuse or Domestic Violence	
History Of Child-hood Abuse	No	58.5
	Unknown	8.2
	Yes	33.3
Physical Abuse	No	72.3
	Unknown	10.1
	Yes	17.6
Sexual Abuse	No	74.8
	Unknown	11.3
	Yes	13.8
Emotional Harm And/Or Neglect	No	66.0
	Unknown	9.4
	Yes	24.5
Substantiated Abuse?	No	72.3
	Unknown	12.6
	Yes	15.1
Domestic Violence	No	72.3
	Unknown	6.9
	Yes	20.8
		
	Precipitating incident or stressful life event	
Precipitating incident or stressful life event	No	9.4
	Yes	90.6
Argument With Significant Other	No	52.8
	Unknown	3.8
	Yes	43.4
Relationship Break-down With	No	67.9
Significant Other	Unknown	2.5
	Yes	29.6
Alcohol, Drug Or Substance Use	No	39.6
	Unknown	6.9
	Yes	53.5
		
	Contagion	
Contagion	No	65.4
	Possibly	10.7
	Yes	23.9
Familial Contagion	No	86.2
	Unknown	.6
	Yes	13.2
Imitative Contagion	No	78.0
	Unknown	3.1
	Yes	18.9
Within 24 Hours Of Another Death	No	86.2
	Unknown	3.1
	Yes	10.7
		
	Child stated and/or implied intent	
Child stated and/or implied intent	No	45.9
	Yes	54.1
Suicide Note	No	79.9
	Unknown	.6
	Yes	19.5

There was a non-significant trend (p=0.080) towards females having a higher SEIFA score (Socio-Economic Indexes for Areas) than males. Males were less likely to be known to the child safety system within the last 3 years before death (p=0.097), have known mental health of Behavioral issues (p=0.032), show previous suicidal thoughts (p=0.014) or attempts (p=0.001) or self-harm (p<0.001), have a history of child abuse (p=0.009) or sexual abuse (p=0.016), and more likely to use infrequent methods such as carbon monoxide poisoning, self-immolation, gunshots, and crashing a vehicle (p=0. 068). In contrast, females more frequently used drug overdoses and jumping from a height as methods of suicide.

Some methods of death (carbon monoxide poisoning, drug overdoses, gunshot wounds, and jumping from a height) were less likely to be associated with previous suicidal thoughts or Behaviors (p<0.001), attempts (p=0.005), self-harm (p=0.019), suicidal ideation (p=0.001), or exposure to domestic violence (p=0.007), perhaps reflecting the low survival rates from these methods. Motor vehicle collisions, carbon monoxide poison, and drug overdoses were less likely to be precipitated by stressful life events (p=0.005). 

Relationship breakdowns were more likely to precipitate hanging, asphyxiation, or lying in front of a moving object. While a history of alcohol, drug or substance use was common, gunshot wounds, self-immolation and carbon monoxide poisoning were more common in non-users (p=0.038). Asphyxiation, carbon monoxide poisoning, and jumping from a height were more likely to be associated with leaving a suicide note (p=0.055). Hanging was the primary method (88%) for young persons who had been influenced by a death within the last 24 hours.

Aboriginal people were over-represented in the data (31%) and were more likely to live in remote or very remote areas (p<0.001), have a very low SEIFA, be known to the child safety system within the last 3 years (p=0.007), have a history of childhood (p=0.060) and physical (p=0.005) abuse, especially substantiated abuse (p=0.001), and been exposed to domestic violence (p=0.004). They were less likely to have known mental health or Behavioral issues (p=0.067). Family contagion, although rare (n=2), and suicide within 24 hours of another death (p=0.064), were more common for Torres Strait Islander families (p=0.022). 

**Qualitative analysis **

Next we present the qualitative analysis of the full data set (n=159), which was conducted using Leximancer. Themes identified within the complete data set included deceased (100% relevance), home (77%), ambulance (31%), day (27%), neck (19%), left (19%) and hospital (3%). The themes and concepts are discussed below and the frequency of concepts is illustrated in Table 2.

**Table 2:** Frequency of concepts revealed through Leximancer analysis of child suicides in Queensland, Australia 2004-2012 (n=159).

Concept	Count	Relevance Percentage		Concept	Count	Relevance Percentage
police	38	9		incident	39	9
deceased	416	100		suicide	34	8
home	133	32		rope	33	8
hanging	115	28		brother	31	7
located	82	20		school	30	7
mother	82	20		called	30	7
ambulance	48	12		night	29	7
day	46	11		tree	28	7
prior	44	11		tied	27	6
bedroom	44	11		life	27	6
family	43	10		attempted	27	6
neck	42	10		child	25	6
left	42	10		bed	24	6
father	42	10		hospital	19	5
friend	42	10		head	17	4
death	40	10				

A key theme identified was the centrality of family. Mothers, fathers, brothers and sisters were frequently involved in incidents prior to and after the suicide attempt. Often, but not always, family were aware of indicators such as changes in Behavior, emotional outbursts, and explicit expressions of suicidal ideation. On occasion, however, the family felt no indication that the child would attempt suicide. In many instances, there was evidence of family conflict prior to the suicide attempt. Mothers were more often mentioned in the cases (count=82) compared with, for example, fathers (count=42) and brothers (count=31). Mothers typically played a broader role, with more interactions with the child, while fathers were more typically involved in checking on, finding the child, and responding to the suicide. 

Similarly, the theme and concept of home was also central to the descriptions. While some suicide attempts took place away from the house, the majority were located in the house with the bedroom, bathroom, garage and shed as frequent locations. Bedrooms featured most strongly as a place of privacy with limited checking from family. In many instances, family had been locked out of the home or room. Trees also featured more heavily than expected in the data (count=28) – both trees near the residence and in public places – as locations for suicide attempts and as part of a hanging attempt. The theme of home overlapped with concepts associated with family, in that it was a frequent location for interpersonal conflict that occurred prior to the suicide attempt.

The themes of day and left reflected the case descriptions’ focus on incidents that had occurred prior to the suicide attempt. For example, the concept prior reflected that events such as interpersonal conflict and arguments (“The deceased was abusive towards both the neighbour and ex-partner and had a knife threatening the ex-partner and threatening self-harm”), romantic relationship breakdown, mental health symptoms, homelessness or home transition, and death of a pet. Events often occurred the night prior to suicide. 

Verbal (“Deceased spoke to his grandfather the afternoon prior to being found and stated that he was going to kill himself. Deceased also wrote a text message to an unknown person stating that he was ‘about to hang’”), written and electronic communication of suicidal ideation, suicidal plans, or messages of affection and farewell, to friends and family were also common. Friends were often aware of unusual Behavior prior to the suicide. Having friends who had also committed suicide was also recurrent in the data (“The deceased had been depressed following the hanging suicide of a fellow student and friend about 3 weeks earlier”.). It was not uncommon on the day of the suicide attempt for the child to appear ‘happy’, ‘peaceful’, ‘normal’ or ‘in good spirits’. 

The theme of neck and also the concept of head referred to methods of suicide. While there were a number of methods of suicide employed, hanging and head injuries (e.g., collisions, jumping from a height or gunshots to the head) were prevalent in the Leximancer analysis. While ropes were common, the wide variety of materials that were tied around the child’s neck during asphyxiation or hanging were broad (e.g., belts, dog chains, electrical cords) reflecting the relative availability and versatility of tools for suicide. 

The themes of ambulance and hospital included concepts such as called, police, bed, life and attempted. Overall, these concepts referred to the post-suicide attempt sequelae of events, where the police and ambulance were often called and signs of life were checked. Some were taken to hospital, which was the final place of death. Others had had previous interactions with the hospital as a result of mental health assessment and treatment, or previous suicide attempts.

A qualitative comparison between hanging and non-hanging suicide was also conducted using Leximancer. Due to the predominance of hanging as a cause of death (n=129; 813% of suicide deaths), the Leximancer analysis for hangings only was very similar to the overall analysis although the new concept of girlfriend was featured, while hospital was not featured (suggesting that these suicides were less likely to result in hospitalisation and more likely to feature romantic relationship stressors). An analysis of non-hanging deaths shows similar considerations to the overall analysis (such as the centrality of family and friends) but also highlights other methods of suicide including collision with a moving vehicle (vehicle, middle, road, stop), gunshots and immolation (heard, loud) and carbon monoxide poisoning. Head wounds featured more strongly (where hanging deaths featured neck injury), and messages to friends were also prominent. Another new concept that emerged was attended, where the deceased was attended normal activities such as school or parties immediately prior to the death.

## Discussion

The analysis of suicide death data for 159 cases revealed some key themes that could be utilised to better understand suicide in youth and to inform prevention efforts. 

Gender differences were seen with the suicide mechanism: only males died by suicide by collision with a moving object (train, car) or carbon monoxide poisoning, and they were more likely to die of a gunshot wound; only females died as a result of an overdose or through asphyxiation with a gas other than carbon monoxide. Similarly, differences existed in the child’s choice of suicide location: females more frequently died by suicide in their bedroom and males more frequently died by suicide in public locations. 

There seems to be increased suicide risk associated with mental health difficulties like depression,^8^ however this could suggest health services/health system failure. Children with mental health difficulties, and their families, need additional support to minimise suicide risk.^2,24,25^ Evidence of the child planning their suicide was apparent in over half of the cases, suggesting that indicators of planning should be identified through additional research, and that members of the community need to be educated that indicators of planning suggest imminent suicide risk. The ‘planning period’ may provide a window of opportunity for prevention. However, family and friends need to be better connected to health services. Failure of the system was evident in the case of one child who suicided the night after a mental health assessment.

Managing mechanisms of death frequently features in injury prevention efforts. In contrast to suicides in other jurisdictions such as the United States,^28^ only 4.4% of cases involved guns. Gun access in Australia was severely restricted by national legislation in 1997, ^29^ contributing to the reduced proportion of suicide fatalities resulting from gunshot wounds; ^3^ a similar trend has been found in gun-related suicides in Canada. ^30^

In Queensland, the proportion of suicides completed by gunshot has mirrored the falling household gun ownership rates, with both currently around 10%. ^29^ However, contention remains regarding mechanism substitution, with suicides increasingly being completed by hanging.^7^ More than three quarters of the child suicides in the current review were by hanging, which is more difficult to control through restriction efforts.^31^

Research demonstrates that young people value family relationships, friendships, being independent, physical and mental health, and that mental health/coping with stress/depression, body image, and family conflict are of high concern.^32^ Accordingly, these factors may be potential indicators of risk of self-harm and can be used to guide injury prevention efforts. ^20^ In addition, children and adolescents most commonly seek advice and support from friends, parents, other relatives, and family friends. ^33^

Importantly for intervention and the identification of risk factors, young people do not live segregated from the stresses of their families and the wider community. As a result they could be subject to various amounts of toxic stress and development of unhealthy coping mechanism (i.e. isolation) that can lead to suicidal ideation and attempts.^13^ Mattila et al. ^34^ reported on the impact of economic depression on suicide amongst adolescents 10-19 years (1990-1991), an increase that was particularly sharp for males 15-19 years. 

In Australia, suicide peaks in males of the same age group were similarly seen during the late 1980s, reportedly a consequence of social isolation and unemployment.^8^ In both cases, the corresponding rates for females were lower, and more stable. ^8,34^ This suggests that financial or employment concerns (among others) traditionally may impact on genders differently, although evidence of this impact was lacking in the case descriptions in the current study. A large portion of the deceased in this study were from disadvantaged backgrounds (as indicated by SEIFA scores), which could suggest that concerns about future prospects may also contribute to suicide even at an early age. In addition to factors that are unique to – or weigh more heavily upon –young people, consideration of the broader societal stressors is important for targeted interventions, particularly for males. 

Options such as free telephone counselling service, online counselling websites, and community agencies, whilst potentially helpful for children who engage with these activities, are not enough, particularly as many young people state they are not comfortable using such services.^33^ Furthermore, in addition to the gaps in health services discussed previously, there appears to be a lack of communication between departments (e.g., schools – staying home from school to suicide) and other groups who play a role in child safety (e.g., police detecting and apprehending for graffiti) and parents. 

Reporting of possible attempted and completed suicides, including consideration of known risk factors, needs to be thorough and applied consistently across all relevant domains (coronial, police and health records) in order for meaningful studies to contribute to future interventions. 

A breadth of future research is required. Within the domain of child mental health, depression, substance abuse, impulse control, coping, accepting help, gender diversity, and sexuality, and their relationship with suicidal-orientation and suicide risk all require further investigation. Given the unique developmental circumstances of childhood and adolescence, integrated injury prevention approaches which can harness the capacities of parents, family and broader networks such as schools are required.^35^ In addition, application of theoretical models of adolescent suicide risk appear warranted and similarly may inform injury prevention. ^13,18,19,36^

## Conclusion

Child suicide is a global problem and effective intervention requires a comprehensive understanding of risk factors and a holistic approach. Additional attention and resources dedicated to this public health issue will considerably enhance the development and implementation of effective intervention strategies targeting child and adolescent suicide. 
